# Effects of feeding high-moisture corn on milk quality, serum antioxidant indices and intestinal microflora of Chinese Holstein cows

**DOI:** 10.3389/fvets.2025.1732794

**Published:** 2026-04-10

**Authors:** Tong Li, Yongkuo Li, Subinur Abduli, Linhai Song, Buweiaizar Maimetimin, Wei Shao, Liang Yang, Yong Wei, Wanping Ren

**Affiliations:** College of Animal Science, Xinjiang Agricultural University, Urumqi, China

**Keywords:** antioxidant capacity, Chinese Holstein dairy cows, high-moisture corn, intestinal microbial flora, milk quality, serum antioxidant indices

## Abstract

**Introduction:**

This study investigated the effects of replacing steam‑flaked corn (SFC) with high‑moisture corn (HMC) in the diet of Chinese Holstein dairy cows.

**Methods:**

Forty mid‑lactation cows (parity 2–3) were randomly assigned to two groups: a control group (YPFY group, *n* = 20) fed a diet containing SFC, and a treatment group (GSFY group, *n* = 20) fed HMC on an equal dry‑matter basis, with both diets being isoenergetic and isonitrogenous.The experiment consisted of a 10-day acclimation period followed by a 60-day feeding trial, during which milk quality, serum antioxidant capacity, and intestinal microbiota were evaluated.

**Results:**

Although milk yield was unaffected, the GSFY group showed a marked increase in milk fat percentage (*p* < 0.01), while lactose, protein, and solids‑not‑fat exhibited non‑significant decreasing trends. Serum antioxidant capacity was significantly enhanced in the GSFY group, as reflected by elevated activities of superoxide dismutase, glutathione peroxidase, and catalase (*p* < 0.05). Gut microbial alpha diversity did not change, but beta diversity revealed significant structural separation. At the phylum level, HMC feeding increased the abundance of Bacteroidota while decreasing *Firmicutes_A* and *Actinobacteriota*. At the genus level, *Paraprevotella* and *Succinivibrio* were enriched, whereas *Prevotella* and *Faecousia* were reduced (*p* < 0.05). Correlation analysis indicated a strong link between these microbial shifts and milk composition.

**Discussion:**

In conclusion, HMC serves as a superior alternative to SFC, improving milk fat, enhancing systemic antioxidant status, and positively modulating the gut microbiota to optimize milk component synthesis in Chinese Holstein cows.

## 1 Introduction

Corn is one of the most crucial raw materials in dairy cows farming and serves as the primary energy source for dairy cows. For the dairy industry, how to enhance the utilization efficiency of corn has consistently been a hot topic in the dairy cows farming sector. HMC is characterized by low toxin levels, high nutritional value, high digestibility, and low feeding costs (1, 2). In recent years, considerable research has been conducted by researchers on the feeding value of HMC in dairy cows nutrition. The U.S. NRC (3) stated that HMC diets can optimize energy partitioning in dairy cows, leading to a 12–15% increase in the proportion of short-chain fatty acids in milk fat. Dai et al. (4) and Zhou et al. (5) demonstrated that the primary reason for the low toxin content in HMC is that lactic acid bacteria produce lactic acid during its fermentation process, which subsequently lowers the pH and inhibits the growth and reproduction of spoilage bacteria. The starch digestibility of HMC in the rumen, small intestine, and throughout the entire gastrointestinal tract is higher than that of SFC and conventional dry corn (6, 7). Han et al. (8) conducted a comparative analysis of data from pastures in northern China and found that while feeding HMC had no significant impact on dry matter intake, milk yield, or milk composition in dairy cows, it could significantly reduce milk production costs, thereby enhancing the economic benefits of the pastures.

It is noteworthy that some nations have implemented large-scale adoption of HMC feeding systems since the 1970s. Currently, 82% of lactating cows farms in the European Union use it as the core energy feed (9), however, relevant research in China is still in its infancy, and further in-depth studies are required to elucidate the regulatory mechanisms of HMC on the health of dairy cows, including its effects on milk yield and milk composition. Therefore, this study utilized Chinese Holstein dairy cows as the research subjects to explore the three-dimensional impacts of feeding HMC on their milk quality (including milk yield and milk composition), antioxidant capacity (measured by the activities of SOD, GSH-PX, and CAT), and intestinal microbiota (evaluated in terms of Alpha/Beta diversity and key functional bacterial genera), and the combined analysis of the dairy quality and intestinal microbiota of Chinese Holstein dairy cows fed with HMC was discussed. In conclusion, this study provides a basis for the scientific application of HMC in dairy cow breeding and the improvement of feed utilization efficiency.

## 2 Materials and methods

### 2.1 Animals

The experiment enrolled 40 healthy Chinese Holstein dairy cows with 60 days in milk and parity between 2 and 3 as experimental subjects. The animal care protocol for this experiment was approved by the Animal Ethics Committee of Xinjiang Agricultural University (Urumqi, China; Approval No: 20240720), and all efforts were made to minimize animal distress.

### 2.2 Time and location

The experiment was conducted at the cows farm of Shawan Tianrun Animal Husbandry Co., Ltd., located in Tacheng Prefecture, Xinjiang Uyghur Autonomous Region. The overall project spanned from September 2024 to December 2024. Initial preparations, including animal selection, grouping, and diet formulation, were completed in September. The core animal trial commenced thereafter, consisting of a 10-day adaptation period followed by a 60-day experimental feeding and data collection period. The samples collected over the 60-day experimental phase were tested and analyzed, with subsequent analysis lasting until December.

### 2.3 Experimental design

Forty healthy mid-lactation Chinese Holstein cows (approximately 60 days in milk, parity 2–3) were selected as experimental subjects. Using a single-factor completely randomized design, the cows were randomly assigned to two dietary treatment groups: the control group (YPFY group, *n* = 20), which was fed a basal total mixed ration containing steam-flaked corn (SFC), and the treatment group (GSFY group, *n* = 20), in which SFC in the diet was replaced with high-moisture corn (HMC) on an equal dry matter basis, and careful formulation adjustments were made to ensure the two diets were isoenergetic and isonitrogenous. Diet formulations were calculated using the AMTS.Cattle.Professional software (AMTS, LLC, USA) (for detailed diet composition, see Table 1). The trial consisted of a 10-day adaptation period followed by a 60-day formal feeding period, during which all cows were maintained under identical housing and management conditions.

**Table 1:** Raw material composition and nutrient level of the diet (DM).

Composition (%)/item	YPFY^1^	GSFY^2^
Silage (%)	49.1	48.4
Dairy concentrate Supplement^3^	19.2	19.0
(%)		
High-moisture corn (%)	0.0	7.8
SFC (%)	6.4	0.0
Alfalfa hay (%)	2.1	2.1
Alfalfa silage (%)	4.3	4.2
Dairy concentrate feed (%)	7.5	7.4
Cottonseed (%)	4.3	4.2
Beet pulp pellets (%)	2.1	2.1
Molasses (%)	3.9	3.8
Sodium bicarbonate (%)	0.4	0.4
Fat powder (%)	0.3	0.3
Fatty acid calcium (%)	0.3	0.3
Total	100	100
Nutrient level		
CP (%)^4^	16.7	16.6
Starch (%)	26.8	26.8
EE(MJ/kg)^4^	4.6	4.6
*NEL* (MJ/kg)^5^	1.7	1.7
ADF (%)^4^	16.5	16.5
NDF (%)^4^	29.0	29.1
peNDF (%)^4^	18.0	17.8

1 YPFY: control group, the diet include 6.41% SFC.

2 GSFY: experimental group, the diet include 7.79% high-moisture corn.

3 Premixes are provided per kilogram of diet. The premix provided the following per kg of diets: VA 200000 IU, VD 40000 IU, VE 2000 IU, Cu 180 mg, Zn 1,000 mg, Mn 800 mg, I 15 mg, Se 10 mg.

4 CP, Crude Protein; EE, Energy Efficiency; ADF, Acid Detergent Fiber; NDF, Neutral Detergent Fiber; peNDF, Physically Effective Neutral Detergent Fiber.

5 NEL: Net Energy for Lactation. The net energy for lactation was calculated with reference to the NRC (2001) (3), while the rest were measured values. NEL was a calculated value according to NRC (2001), while the others were measured values.

### 2.4 Feeding and management of experimental animals

All the experimental cows were raised in the same pen in a scattered manner. They were fed three times a day at 9:00, 13:00, and 20:00 respectively, and milked three times a day at 8:30, 12:30, and 19:30. During this period, they could ad libitum access to feed and water. The diet formulations and nutritional levels for the experimental cows were detailed in Table 1.

### 2.5 Sample collection and indicator measurement

#### 2.5.1 Milk sample collection and indicator measurement

Milking was performed using a fully automatic parallel milking machine at three fixed time points daily (8:30, 12:30, and 19:30), and the individual milk yield of each experimental cow was recorded at each milking session during the experiment period. To ensure the representativeness of milk components, equal volumes of milk samples (50 mL per session) were collected from each cow at each milking, and the three subsamples from the same cow were thoroughly mixed to form a composite daily sample. The components of raw milk, such as milk fat, lactose, milk protein, and non-fat milk solids, were determined using a milk analyzer (Lactoscan Milk Analyzer, Model: MCC-CA-018835, Milkotronic Ltd., Bulgaria) at the Dairy and Meat Analysis Laboratory of Xinjiang Agricultural University. The methods followed the national standard (GB 19301–2010 “National Food Safety Standard for Raw Milk”).

#### 2.5.2 Blood sample collection and indicator measurement

On the 0th, 20th, 40th, and 60th days of the experiment, blood samples were collected from the cows. Before morning feeding, 10 mL of blood was drawn from the coccygeal vein. The blood samples were centrifuged at 3,500 r/min for 10 min to separate the serum. The separated serum was stored at −20 °C for the determination of serum indices. The serum antioxidant indices, including superoxide dismutase (SOD), glutathione peroxidase (GSH-PX), and catalase (CAT), were sent to Beijing Huaying Biotechnology Institute (Beijing, China) for detection on our behalf.

#### 2.5.3 Fecal sample collection and indicator measurement

Prior to the morning feeding on day 60 of the experiment, 500 g of fecal samples from the hindgut were collected using the rectal sampling method. The samples were subpackaged and cryopreserved for subsequent determination of intestinal microbiota (10). The genomic DNA extraction, PCR amplification, library construction, and sequencing for the determination of intestinal microbiota indices were all conducted by Hangzhou Jingsen Biotechnology Co., Ltd. (Hangzhou, China) on our behalf. The hypervariable V3-V4 region of the bacterial 16S rRNA gene was selected for sequencing. PCR amplification was performed using the universal primer sequences 338F (5′-ACTCCTACGGGAGGCAGCA-3′) and 806R (5′-GGACTACHVGGGTWTCTAAT-3′), raw image data obtained via high-throughput sequencing were converted into raw sequencing reads through base calling analysis (11). Alpha diversity indices (including Chao1 index, Shannon index, and Simpson index) and Beta diversity were calculated using the QIIME software (Rob Knight Lab, Boulder, Colorado), species annotation of the feature sequences was conducted using QIIME2 software (Biome Software Inc., Denver, Arizona) in conjunction with the SILVA database, and biological classification was performed at the levels of domain, phylum, class, order, family, and genus (12, 13).

### 2.6 Statistical analysis

The data of milk quality and serum antioxidant index were preliminarily processed using Excel 2016 (Microsoft Corp., Redmond, WA, USA) and analyzed for variance using SPSS 27.0 statistical software (software version 20.0, IBM, NY), the independent samples *t*-test method was used for comparison between groups. *p* < 0.05 indicated a significant difference, and *p* > 0.05 indicated no significant difference. The test results were all expressed as the Mean and the mean standard deviation (Mean ± SD).

The analytical methods for 16S rRNA gene sequencing data were as follows: data were analyzed in QIIME2, Alpha diversity was compared via (*t*-test, *p* < 0.05); Beta diversity was assessed using PCoA based on Bray-Curtis distances; sequences were clustered into OTUs at 97% similarity; taxonomy was annotated against the SILVA database; relative abundances at the phylum/genus levels were analyzed (*t*-test, *p* < 0.05); the top 10 taxa were shown; the heatmap displayed normalized, clustered metabolic patterns.

The analytical method for the correlation between gut microbiota and milk components was as follows: by calculating Pearson correlation coefficients between the relative abundance of bacterial genera and key milk parameters, statistical significance of the correlations was assessed, and the resulting correlation matrix was visualized as a heatmap.

## 3 Results

### 3.1 Effects of feeding HMC on milk quality of Chinese Holstein dairy cows

#### 3.1.1 Effects of feeding HMC on lactation performance of Chinese Holstein dairy cows

The effects of feeding HMC on lactation performance of Chinese Holstein dairy cows were shown in Table 2. On the 20th and 60th days, the milk yield of the GSFY group decreased by 0.56 and 0.06%, respectively, compared to the YPFY group (*p* > 0.05), both groups exhibited a declining trend in milk yield; the lactose rate of GSFY group was 0.68 and 2.21% lower than that of YPFY group on 20 d and 60 d, respectively (*p* > 0.05); milk fat percentage increased by 3.49, 4.42, and 10.79% (*p* < 0.01) in GSFY group compared to YPFY group on 20 days, 40 days, and 60 days, respectively; the milk protein rate was 0.92, 0.40, and 2.07% lower in GSFY group compared to YPFY group on 20 d, 40 d, and 60 d, respectively, in both groups (*p* > 0.05); the non-dairy fat solids content was 1.44 and 5.21% lower in GSFY group compared to YPFY group on 20 and 60 days, respectively, in both groups (*p* > 0.05).

**Table 2:** Effect of feeding HMC on milk yield and milk composition in Chinese Holstein cows.

Items	Time	YPFY^1^	GSFY^2^	*p*-value
Milk yield (kg)	0 d	44.20 ± 0.89	44.47 ± 0.72	0.97
	20 d	41.29 ± 0.98	41.52 ± 0.83	
	40 d	38.45 ± 0.66	38.39 ± 0.61	
	60 d	35.36 ± 0.54	35.38 ± 0.45	
Lactose (%)	0 d	4.84 ± 0.12	4.89 ± 0.10	0.49
	20 d	4.87 ± 0.16	4.84 ± 0.11	
	40 d	4.91 ± 0.11	4.97 ± 0.26	
	60 d	5.05 ± 0.30	4.94 ± 0.15	
Milk fat (%)	0 d	3.38 ± 0.29	3.34 ± 0.24	< 0.01
	20 d	3.58 ± 0.23^b^	3.71 ± 0.12^a^	
	40 d	3.39 ± 0.20^B^	3.54 ± 0.17^A^	
	60 d	3.52 ± 0.31^B^	3.90 ± 0.17^A^	
Milk protein (%)	0 d	3.32 ± 0.11	3.29 ± 0.07	0.16
	20 d	3.23 ± 0.15	3.22 ± 0.07	
	40 d	3.28 ± 0.09	3.27 ± 0.16	
	60 d	3.37 ± 0.21	3.28 ± 0.09	
Non-fat solids (%)	0 d	8.60 ± 0.26	8.65 ± 0.21	0.10
	20 d	8.86 ± 0.41	8.73 ± 0.21	
	40 d	8.90 ± 0.24	8.97 ± 0.42	
	60 d	9.46 ± 0.75	9.08 ± 0.34	

1 YPFY: control group, the diet include 6.41% SFC.

2 GSFY: experimental group, the diet include 7.79% high-moisture corn.

Within the same group of items, for data in the same row, ^ab^ different lowercase superscripts indicate significant differences (*P* < 0.05), ^AB^ different uppercase superscripts indicate extremely significant differences (*P* < 0.01).

#### 3.1.2 Effect of feeding HMC on amino acids in the milk of Chinese Holstein cows

The effects of feeding HMC on amino acids in the milk of Chinese Holstein dairy cows were shown in Table 3. The glycine, isoleucine, tryptophan, methionine, phenylalanine, proline, and tyrosine contents in GSFY group cow’s milk were increased by 49.20, 72.85, 31.59, 21.32, 59.97, 33.01, and 91.96%, respectively, as compared to YPFY group (*p* < 0.01); the glutamine, leucine, lysine, and threonine contents of GSFY group were reduced by 28.12, 14.20, 51.15, and 33.01%, respectively, compared with YPFY (*p* < 0.05).

**Table 3:** Effect of feeding HMC on amino acids in the milk of Chinese Holstein cows.

Items	YPFY^1^	GSFY^2^	*P*-value
Alanine (ng/mg)	50069.60 ± 3629.00	45730.27 ± 3874.24	0.06
Arginine (ng/mg)	7414.54 ± 635.93	7003.60 ± 854.77	0.08
Asparagine Anhydrous (ng/mg)	1879.50 ± 201.80	1789.49 ± 195.64	0.07
AsparticAcid (ng/mg)	1554.69 ± 208.45	1524.84 ± 160.01	0.97
Glutamine (ng/mg)	2078.87 ± 297.57^a^	1494.39 ± 191.08^b^	0.03
GlutamicAcid (ng/mg)	9223.37 ± 804.88	9223.37 ± 880.07	0.99
Glycine (ng/mg)	2512.27 ± 305.32^B^	3748.22 ± 121.08^A^	< 0.01
Histidine (ng/mg)	2111.82 ± 231.58	2326.65 ± 155.54	0.07
Isoleucine (ng/mg)	486.84 ± 56.31^B^	841.51 ± 92.81^A^	< 0.01
Leucine (ng/mg)	8402.45 ± 620.17^a^	7209.68 ± 511.54^b^	0.04
Hydroxyproline (ng/mg)	2830.19 ± 283.49	2496.75 ± 225.71	0.47
Tryptophan (ng/mg)	252.94 ± 23.32^b^	332.84 ± 21.20^a^	0.03
Lysine (ng/mg)	7139.63 ± 640.05^A^	3487.97 ± 289.57^B^	< 0.01
Methionine (ng/mg)	422.96 ± 30.23^b^	513.14 ± 44.91^a^	0.04
Phenylalanine (ng/mg)	361.24 ± 31.14^b^	577.84 ± 44.93^a^	0.02
Proline (ng/mg)	3339.93 ± 291.78^b^	4442.20 ± 319.05^a^	0.03
Serine (ng/mg)	1929.46 ± 151.39	2121.04 ± 136.15	0.77
Threonine (ng/mg)	395.09 ± 25.72^a^	264.73 ± 20.33^b^	0.04
Tyrosine (ng/mg)	697.50 ± 53.82^B^	1338.86 ± 163.80^A^	< 0.01
Valine (ng/mg)	3519.34 ± 263.44	3806.05 ± 287.01	0.37

1 YPFY: control group, the diet include 6.41% SFC.

2 GSFY: experimental group, the diet include 7.79% high-moisture corn.

For data in the same row, ^ab^ different lowercase superscripts indicate significant differences (*p* < 0.05); ^AB^ different uppercase superscripts indicate extremely significant differences (*p* < 0.01).

#### 3.1.3 Effect of feeding HMC on fatty acids in milk of Chinese Holstein cows

The effect of feeding HMC on fatty acids in milk of Chinese Holstein cows were shown in Table 4. The methyl caprylate, methyl decanoate, methyl undecanoate, methyl laurate, methyl tridecanoate, methyl myristate, and methyl pentadecanoate were increased by 89.31, 24.48, 29.24, 27.28, 44.01, 24.77, and 37.54%, respectively, in the milk of GSFY group dairy cows compared with that of YPFY group (*p* < 0.05), the fatty acid contents of methyl palmitate, methyl palmitoleate, methyl heptadecanoate, methyl stearate, methyl trans-isoleate, methyl linoleate, methyl arachidonate, and arachidonic acid were increased by 18.61, 23.90, 41.75, 18.20, 31.32, 22.69, 20.64, and 27.61%, respectively (*p* < 0.05).

**Table 4:** Effect of feeding HMC on fatty acids in milk of Chinese Holstein cows.

Items	YPFY^1^	GSFY^2^	*P*-value
Methyl Caproate (μg/mL)	253.68 ± 17.22	288.54 ± 17.12	0.06
Methyl Caprylate (μg/mL)	243.71 ± 17.59^B^	461.38 ± 23.41^A^	<0.01
Methyl Caprate (μg/mL)	513.84 ± 49.62^b^	639.63 ± 67.49^a^	0.04
Methyl Undecanoate (μg/mL)	23.80 ± 2.85^b^	30.76 ± 2.78^a^	0.03
Methyl Laurate (μg/mL)	680.13 ± 33.63^b^	865.68 ± 62.07^a^	0.03
Methyl Tridecanoate (μg/mL)	20.29 ± 1.58^b^	29.23 ± 1.98^a^	0.02
Methyl Myristate (μg/mL)	1923.10 ± 27.90^b^	2399.51 ± 20.25^a^	0.03
Methyl Myristoleate (μg/mL)	65.28 ± 4.91	69.13 ± 5.34	0.08
Methyl Pentadecanoate (μg/mL)	175.71 ± 15.89^b^	241.67 ± 11.68^a^	0.03
Methyl Palmitate (μg/mL)	5050.03 ± 270.47^b^	5989.96 ± 174.73^a^	0.04
Methyl Palmitoleate (μg/mL)	220.40 ± 11.33^b^	273.08 ± 11.70^a^	0.04
Methyl Heptadecanoate (μg/mL)	54.13 ± 15.16^b^	76.73 ± 19.83^a^	0.02
Methyl Stearate (μg/mL)	1837.71 ± 133.75^b^	2172.21 ± 190.56^a^	0.04
Methyl Trans-vaccenate (μg/mL)	110.03 ± 7.94^b^	144.49 ± 12.39^a^	0.03
Methyl Oleate (μg/mL)	2617.15 ± 21.30	2972.68 ± 27.86	0.24
Methyl 10-Transnonadecenoate (μg/mL)	19.32 ± 1.76	17.04 ± 1.84	0.07
Methyl Linoleate (μg/mL)	361.91 ± 25.31^b^	444.01 ± 20.48^a^	0.03
Methyl Arachidate (μg/mL)	12.50 ± 1.98^b^	15.09 ± 2.02^a^	0.04
Methyl Gamma Linolenate (μg/mL)	4.81 ± 0.78	4.99 ± 0.61	0.85
Methyl Alpha Linolenate (μg/mL)	15.49 ± 5.40	17.48 ± 3.89	0.07
Methyl Heneicosanoate (μg/mL)	9.63 ± 0.73	10.03 ± 0.66	0.09
Methyl 11–14 Eicosadienoate (μg/mL)	4.44 ± 0.56	4.63 ± 0.47	0.61
Methyl Behenate (μg/mL)	6.59 ± 0.82	6.73 ± 0.72	0.18
Methyl Homogamma Linolenate (μg/mL)	10.22 ± 3.09	11.36 ± 2.37	0.38
Methyl Tricosanoate (μg/mL)	2.73 ± 0.20	2.73 ± 0.19	0.99
Methyl Arachidonate (μg/mL)	12.03 ± 3.60^b^	15.36 ± 3.88^a^	0.04
Methyl Lignocerate (μg/mL)	2.15 ± 0.37	2.25 ± 0.38	0.35
Methyl Docosatetraenoate (μg/mL)	11.74 ± 0.50	12.20 ± 0.65	0.59
all-cis-4,7,10,13,16-Docosapentaenoic acid (μg/mL)	3.08 ± 0.43	3.52 ± 0.49	0.06

1 YPFY: control group, the diet include SFC.

2 GSFY: experimental group, the diet include HMC.

For data in the same row, ^ab^ different lowercase superscripts indicate significant differences (*P* < 0.05); ^AB^ different uppercase superscripts indicate extremely significant differences (*P* < 0.01).

### 3.2 Effect of feeding HMC on serum antioxidant indexes of Chinese Holstein cows

The Effect of feeding HMC on serum antioxidant indexes of Chinese Holstein cows were shown in Table 5. It can be seen that the superoxide dismutase activity in the serum of the two groups of cows was increased by 14.98, 7.87, and 7.86% in GSFY group compared with that of YPFY group on the 20th, 40th, and 60th days, respectively (*p* < 0.05), serum superoxide dismutase levels tended to increase in both groups; the serum glutathione peroxidase activity in cows of both groups was increased by 6.17, 6.73, and 4.32% (*p* < 0.05) in GSFY group compared to YPFY group on the 20th,40th, and 60th d, respectively, and there was a tendency for serum glutathione peroxidase levels to increase in both groups; the serum peroxidase activity in the serum of cows in the two groups increased by 7.96, 14.33, and 12.63% (*p* < 0.05) on the 20 d, 40 d, and 60 d in GSFY group compared with that of YPFY group, respectively, and there was an increasing trend in the serum peroxidase levels in the two groups.

**Table 5:** Effect of feeding HMC on serum antioxidant indexes of Chinese Holstein cows.

Item	Time	YPFY^1^	GSFY^2^	*P*-value
SOD^3^(U/ml)	0 d	64.10 ± 0.86	69.51 ± 0.89	0.02
	20 d	66.09 ± 1.21^b^	75.99 ± 1.33^a^	
	40 d	82.82 ± 1.06^b^	89.34 ± 1.50^a^	
	60 d	75.53 ± 2.32^b^	81.47 ± 1.82^a^	
GSH-PX^4^(U/ml)	0 d	134.30 ± 4.65	145.07 ± 1.93	0.02
	20 d	141.84 ± 1.75^b^	150.59 ± 1.71^a^	
	40 d	155.38 ± 2.32^b^	165.83 ± 1.57^a^	
	60 d	152.87 ± 1.80^b^	159.47 ± 3.33^a^	
CAT^5^(U/ml)	0 d	32.85 ± 1.3	36.75 ± 1.30	0.03
	20 d	35.29 ± 1.62b	38.10 ± 1.80a	
	40 d	40.97 ± 0.75b	46.84 ± 1.18a	
	60 d	37.04 ± 1.15b	41.72 ± 1.03a	

1 YPFY: control group, the diet include SFC.

2 GSFY: experimental group, the diet include HMC.

3 SOD: Superoxide Dismutase.

4 GSH-PX: Glutathione Peroxidase.

5 CAT: Catalase.

Within the same group of items, for data in the same row. ^ab^ Different lowercase superscripts indicate significant differences (*P* < 0.05).

### 3.3 Effect of feeding HMC on intestinal microflora of Chinese Holstein cows

#### 3.3.1 Alpha diversity analysis

**Figure 1:**
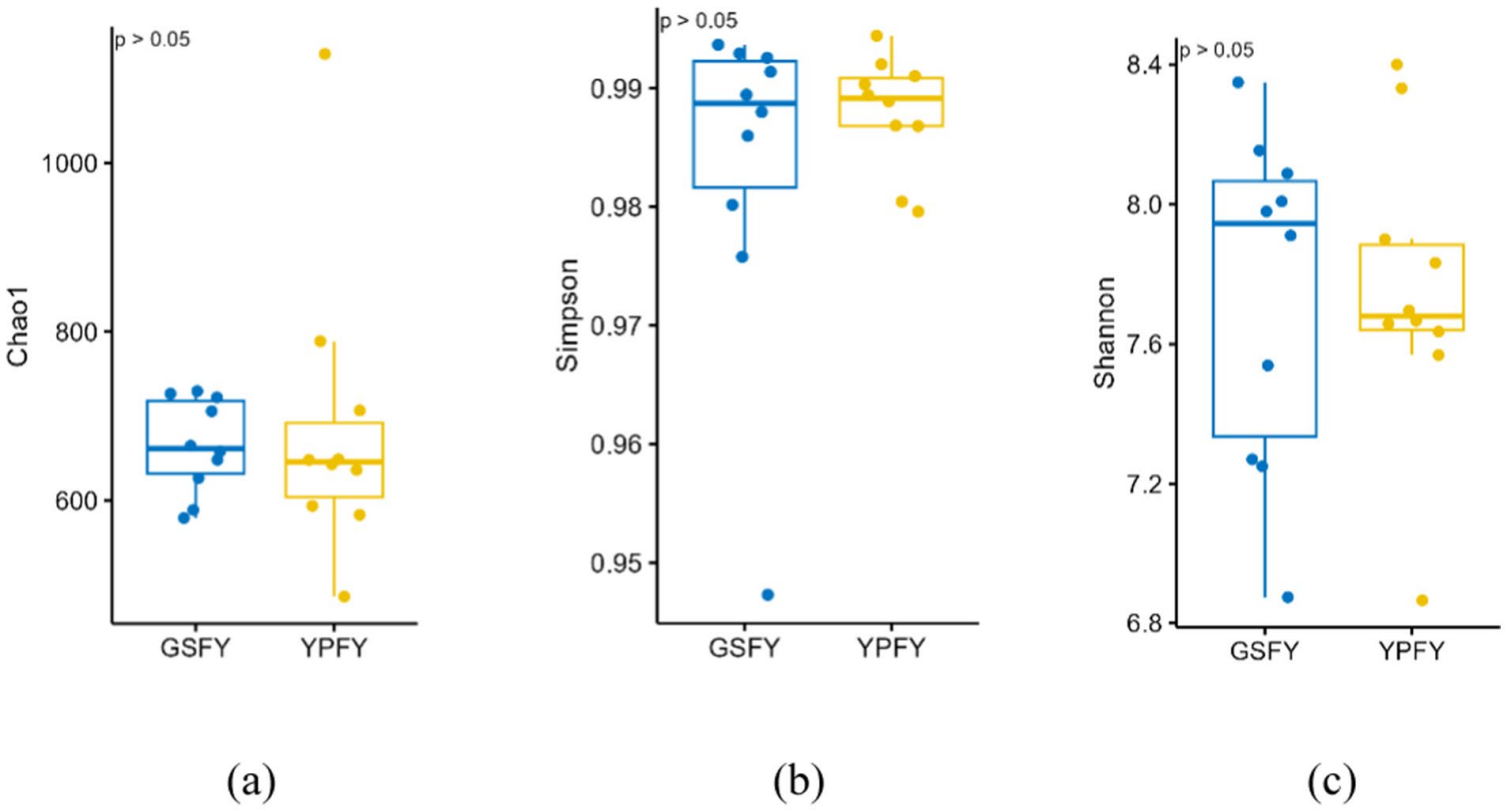
Effect of feeding HMC on the alpha diversity of intestinal microorganisms in Chinese Holstein cows. YPFY: control group, the diet include 6.41% SFC; GSFY: experimental group, the diet include 7.79% high-moisture corn. **(a)** Chao1 index: it is used to estimate the total number of species in a community, mainly focusing on reflecting species richness. The larger the value, the more potentially undiscovered species there may be, that is, the higher the species richness of the community. **(b)** Simpson index: it comprehensively reflects species richness and evenness. The closer its value is to 1, the more species are not only rich in the community, but also the more evenly the distribution of individual numbers of each species is, and the dominant species are not prominent. **(c)** Shannon index: it takes into account species richness and the evenness of each species. The larger the value, the more species there are in the community, the more uniform the distribution of individuals, the greater the uncertainty, and the higher the diversity.

The effect of feeding HMC on the Alpha diversity of intestinal microorganisms in Chinese Holstein cows was shown in Figure 1, and the Chao1 index of GSFY group (665.05) was slightly lower than that of YPFY group (686.44) (*p* > 0.05), with a difference of 3.12%; the Shannon index of GSFY group (7.74) was slightly lower (*p* > 0.05) compared to YPFY group (7.76) with a difference of 0.18%; the Simpson’s index of GSFY group (0.9837) was slightly lower than that of YPFY group (0.9880) (*p* > 0.05), with a difference of 0.43%.

#### 3.3.2 Beta diversity analysis

**Figure 2:**
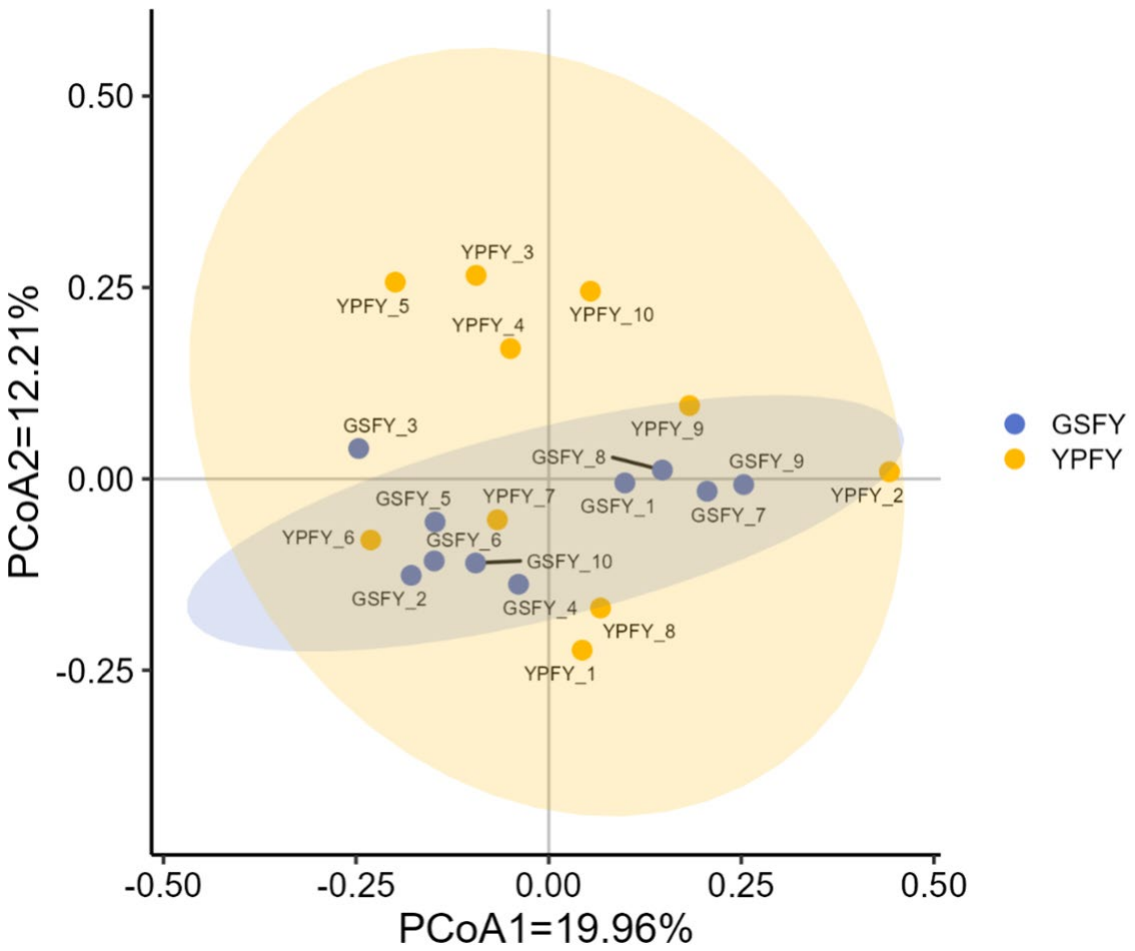
Effect of feeding HMC on the Beta diversity of intestinal microorganisms in Chinese Holstein cows. YPFY_1–10: Ten samples were randomly selected from the YPFY group as subjects for this assay. GSFY_1–10: Ten samples were randomly selected from the GSFY group as subjects for this assay.

The effects of feeding HMC on the Beta diversity of intestinal microorganisms in Chinese Holstein cows were shown in Figure 2, explanation rate of gut microbiota PCoA1 = 19.96% and PCoA2 = 12.21% (total explanation rate 32.17%) for GSFY group vs. YPFY group, each of the two groups of samples were clustered, with a certain tendency to segregate, which shows that the two groups of samples were significantly different based on the structure of the gut microbiota.

#### 3.3.3 OTU analysis

**Figure 3:**
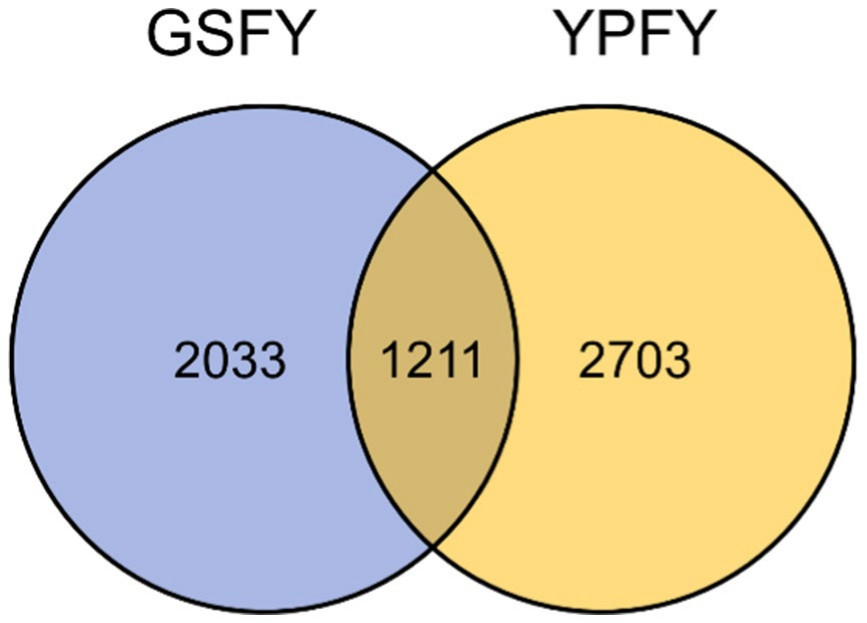
Sample OTUs chart. YPFY: control group, the diet include 6.41% SFC; GSFY: experimental group, the diet include 7.79% HMC.

The results showed that the YPFY group contains 3,914 OTUs and GSFY group contains 3,244 OTUs; the core OTUs between the two were 1,211, with 2,703 unique to YPFY group and 2033 unique to GSFY group; core OTUs (1,211) represent 30.94% of total OTUs in YPFY group and 37.33% in GSFY group (see Figure 3).

#### 3.3.4 Effect of HMC on the relative abundance of intestinal microflora phylum and genus levels in Chinese Holstein cows

**Figure 4:**
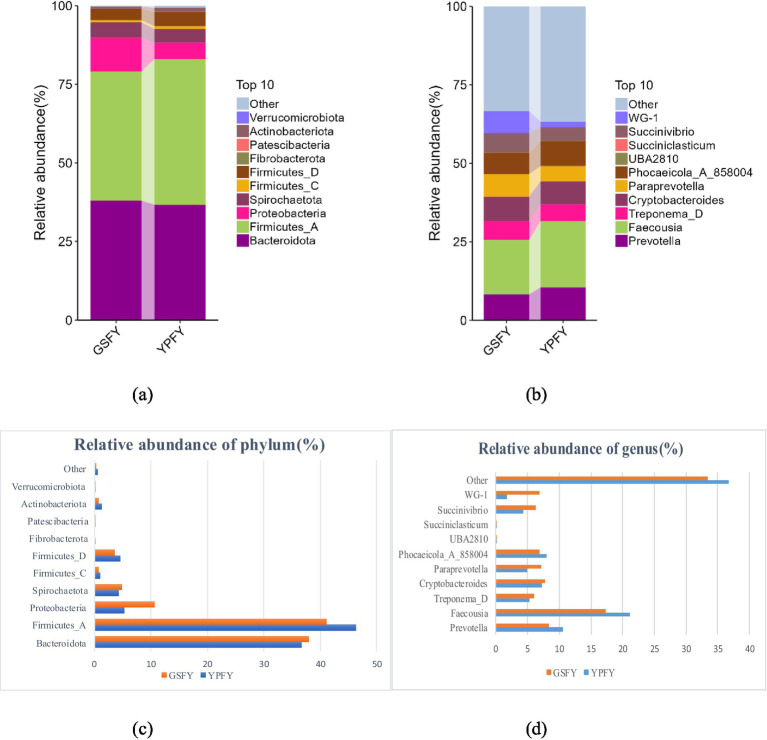
Effect of HMC on the relative abundance of intestinal microflora phylum and genus levels in Chinese Holstein cows. YPFY: Control group, the diet include 6.41% SFC; GSFY: experimental group, the diet include 7.79% HMC. **(a)** The top 10 most abundant gut microbial phyla based on relative abundance at the phylum level. **(b)** The top 10 most abundant gut microbial genera based on relative abundance at the genus level. **(c)** The relative abundances of 10 specific microorganisms at the phylum level. **(d)** The relative abundances of 10 specific microorganisms at the genus level.

The effect of HMC on the relative abundance of intestinal microflora phylum and genus levels in Chinese Holstein cows were shown in Figure 4. The top 10 microbial phyla in terms of relative abundance at the phylum level (Figures 4a,c) and genus level (Figures 4b,d) of the intestinal tract of Chinese Holstein cows can be identified. At the phylum level (Figures 4a,c) *Bacteroidota*, *Firmicutes_A*, and *Actinobacteriota* are dominant phyla of intestinal microorganisms, accounted for 38.01, 41.11, and 0.66% of the relative abundance in GSFY group, respectively, with a significant (*p* < 0.05) increase in *Bacteroidota* in GSFY group compared to YPFY group, while *Firmicutes_A* and *Actinobacteriota* were significantly reduced (*p* < 0.05). At the genus level (Figures 4b,d) *Paraprevotella*, *Succinivibrio*, and *WG-1* are the dominant genera of enteric microorganisms, it accounted for 7.10, 6.22, and 6.91% of the relative abundance in GSFY, respectively, and all three genera were significantly increased in GSFY group compared to YPFY group (*p* < 0.05); at the genus level (Figures 4b,d) *Prevotella* and *Faecousia* accounted for 8.31 and 17.34% of the relative abundance in the GSFY group, respectively, and both genera were significantly reduced (*p* < 0.05) compared to the YPFY group.

#### 3.3.5 Heat map analysis of clustering of differential metabolites of intestinal microorganisms in Chinese Holstein cows fed HMC

**Figure 5:**
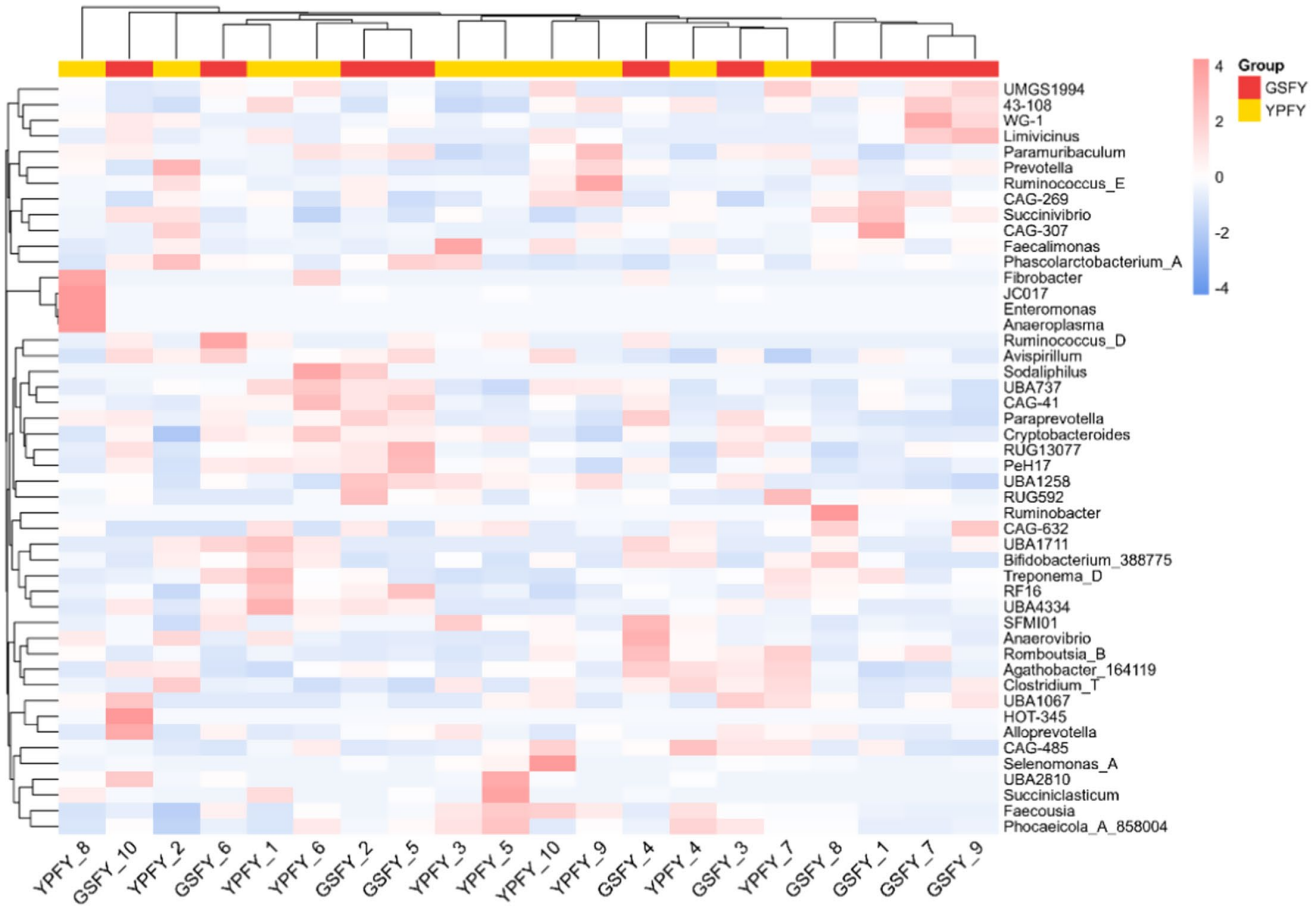
Heat map analysis of clustering of differential metabolites of intestinal microorganisms in Chinese Holstein cows fed HMC. YPFY: Control group, the diet include 6.41% SFC; GSFY: experimental group, the diet include 7.79% HMC. YPFY_1–10: Ten samples were randomly selected from the YPFY group as subjects for this assay. GSFY_1–10: Ten samples were randomly selected from the GSFY group as subjects for this assay.

The clustered heat map of differential metabolite clustering of intestinal microorganisms of Chinese Holstein cows fed HMC was shown in Figure 5, with the sample information in the horizontal direction and the differential metabolite information in the vertical direction, and the different colors represent the different values obtained by normalizing the different relative contents (red for high content and blue for low content). From this, it can be concluded that compared with YPFY group, the metabolic activities of bacterial species such as *Ruminobacter* and *Succinivibrio*, in GSFY group are significantly enhanced; meanwhile, the metabolic activities of bacterial genera such as *Prevotella* and *Faecousia* were significantly decreased (see Figure 5).

### 3.4 Correlation analysis of feeding HMC on intestinal microorganisms and milk composition in Chinese Holstein cows

**Figure 6:**
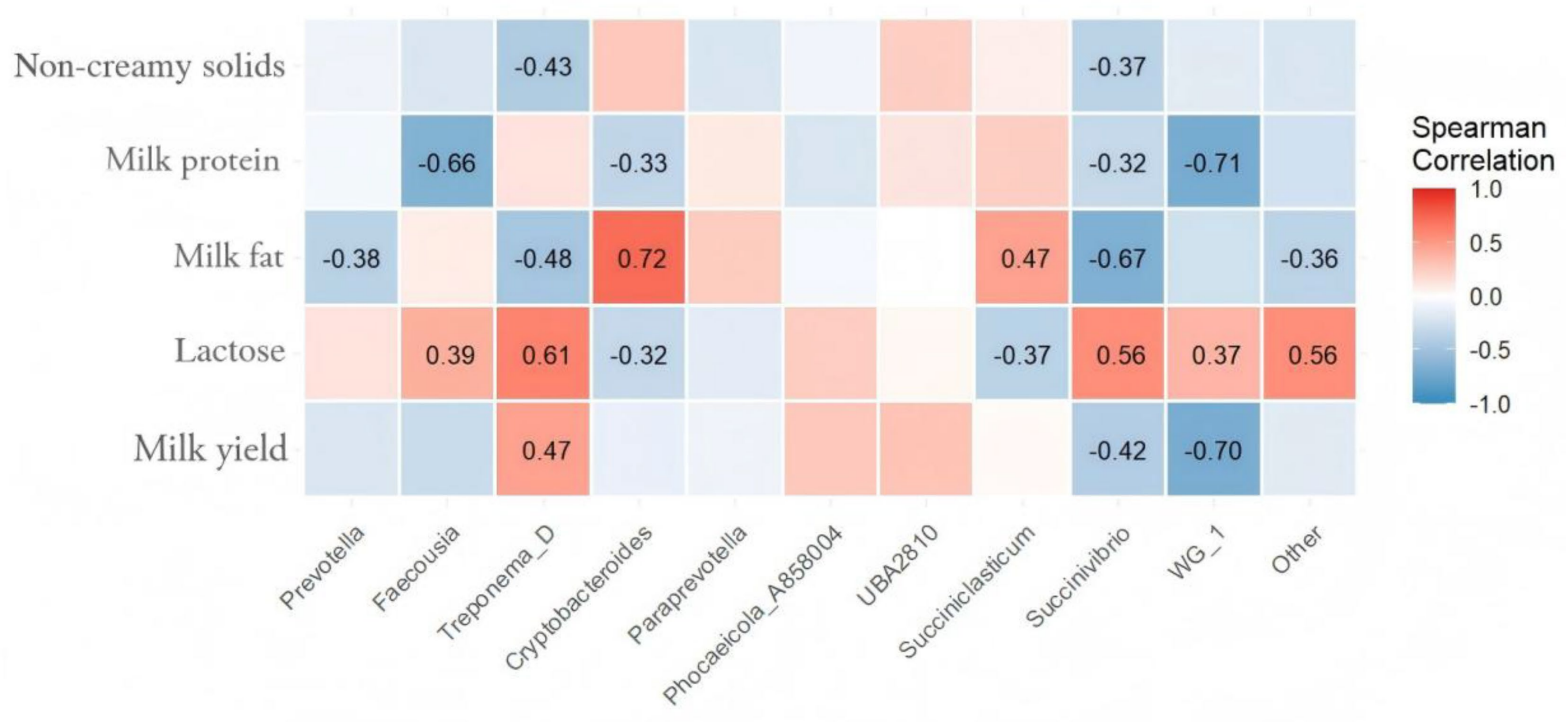
Correlation heatmap showing the relationship between gut microbiota (genus level) and milk composition in Chinese Holstein cows.

The results showed that milk yield was negatively correlated with *Succinivibrio* (*r* = 0.42) and the difference was significant (*p* > 0.05), while it was positively correlated with *Treponema_D* (*r* = 0.47) and the difference was significant (*p* > 0.05); lactose was negatively correlated with *Cryptobacteroides* (*r* = 0.32), *Succiniclasticum* (*r* = 0.37) and the difference was significant (*p* > 0.05), while it was negatively correlated with *Faecousia* (*r* = 0.39), *Treponema_D* (*r* = 0.61), *Succinivibrio* (*r* = 0.56), and *WG_1* (*r* = 0.37) were positively correlated with each other and the difference was significant (*p* > 0.05); milk fat was negatively correlated with *Prevotella* (*r* = 0.38), *Treponema_D* (*r* = 0.48), *Succinivibrio* (*r* = 0.67) and the difference was significant (*p* > 0.05), while it was positively correlated with *Cryptobactereroides* (*r* = 0.72), *Succiniclasticum* (*r* = 0.47) were positively correlated and the difference was significant (*p* > 0.05); milk protein were negatively correlated with *Faecousia* (*r* = 0.66), *Cryptobacteroides* (*r* = 0.33), *Succinivibrio* (*r* = 0.32), and *WG_1* (*r* = 0.71), and the difference was significant (*p* > 0.05); non-creamy solids were negatively correlated with *Treponema_D* (*r* = 0.43) and *Succinivibrio* (*r* = 0.37), and the difference was significant (*p* > 0.05) (see Figure 6).

## 4 Discussion and analysis

### 4.1 Effect of feeding HMC on milk quality of Chinese Holstein cows

Feeding Chinese Holstein cows with HMC in place of SFC maintained milk yield, which agrees with Eun et al. (14) who reported no significant effect on milk production after complete substitution with HMC, its higher moisture content and improved palatability increase dry matter intake (DMI). Moreover, the added moisture softens the feed, raising nutrient digestibility, these changes promote the growth and activity of beneficial microbes, thereby improving ruminal fermentation efficiency (15). In the results of this experiment both groups showed a decreasing trend in milk yield, that are related to the season and the lactation cycle of dairy cows. The contents of lactose, milk protein and non-milk fat solids in GSFY group were slightly lower than those in YPFY group, but the differences between the two groups were not significant. In this experiment, lactose and milk protein in the GSFY group were slightly lower than in the YPFY group, but milk fat content was markedly higher. Yi et al. (16) suggested that the low pH and high nutrient degradability of HMC may cause ruminal metabolic disorders and reduce milk fat percentage, whereas Chandler et al. (17) demonstrated that HMC significantly increased milk fat percentage, which is consistent with our results.

Glycine, L-isoleucine, L-tryptophan and L-tyrosine content in GSFY group milk increased significantly. Shang et al. (18) confirmed that propionate and butyrate produced by HMC fermentation activate the mTOR pathway in bovine mammary epithelial cells, thereby enhancing the transport efficiency of glycine and tyrosine into milk, feeding HMC alters the microbial community by increasing the abundance of *Ruminococcus* and *Lachnospiraceae*, which indirectly regulating amino acid metabolic pathways. Itzhak et al. (19) indicated that the proliferation of *Ruminococcus*, *Lachnospiraceae* and other flora may enhance fiber degradation capacity, produce *α*-ketoacids as carbon skeletons for amino acid synthesis, and thus provide more precursors to promote the synthesis of glycine and L-isoleucine. Conversely, significant reductions were observed in L-glutamine, L-lysine, and L-threonine concentrations. Russell et al. (20) demonstrated that low pH environment may inhibit the activity of certain microorganisms (e.g., *Succinivibrio*), the low pH compromises the integrity of the bacterial cell membrane and inhibits the activity of glutamate dehydrogenase, thereby reducing the synthesis of L-glutamine and L-lysine. Fan et al. (21) stated that HMC high starch digestibility and rapid fermentation increase ammonia nitrogen levels in dairy cows, causing microorganisms to prefer using ammonia nitrogen for microbial protein synthesis rather than converting it into amino acids like threonine. In conclusion, although the overall milk protein rate in the GSFY group showed no significant change, the significant increases in specific amino acids contents suggest that HMC may reshape the nutritional properties of milk protein in milk through microbial metabolism.

The GSFY group showed significantly increased levels of 15 fatty acids in milk, including methyl caprylate and methyl caprate. Voelker et al. (22) demonstrated that ruminal propionate concentration was significantly higher, and propionate is the key substrate for mammary gland to synthesize medium-chain fatty acids, resulting in increased contents of methyl caprylate, methyl caprate, methyl laurate et al.; the fermentation characteristics of HMC can enrich propionate-producing bacteria and fiber-decomposing bacteria in rumen, thus promoting the synthesis of odd-chain fatty acids (OBCFAs). Shang et al. (23) concluded that the concentration of total volatile fatty acids (VFA) in rumen of HMC group was higher, and the abundance of *Prevotella* and other flora in HMC group was also higher, these microorganisms generate propionyl-CoA through *α*-oxidation pathway, and then synthesize odd-chain fatty acids such as methyl undecanoate, methyl tridecanoate and methyl pentadecanoate (24). Rojas-Garduño et al. (25) found that the content of long-chain monounsaturated fatty acids in the milk of the HMC group was significantly increased, which is associated with reduced rumen pH inhibiting the activity of hydrogenating bacteria. In summary, replacing SFC with HMC in the diet of Chinese Holstein cows can increase milk fat percentage.

### 4.2 Effect of feeding HMC on serum antioxidant indices of Chinese Holstein cows

Maintaining oxidative balance in dairy cows supports normal physiological functions (26). Results of this experiment show that feeding HMC significantly enhances serum antioxidant capacity in Chinese Holstein cows compared to SFC, consistent with findings by Shang et al. (18). Ighodaro et al. (27) confirmed that superoxide dismutase and glutathione peroxidase are key antioxidant enzymes, superoxide dismutase first converts superoxide radicals into oxygen and hydrogen peroxide (28), and glutathione peroxidase then decomposes hydrogen peroxide into water and oxygen, protecting cells from oxidative stress damage (29). Albornoz et al. (30) reported that diets with 22% or 28% starch content had no significant effect on dairy cows serum antioxidant indices, however, in this experiment, serum antioxidant indices of GSFY cows were significantly at 20d, 40d and 60d, possibly related to the antioxidant capacity of abundant lactic acid bacteria in HMC (31). In conclusion, feeding HMC can better improve serum antioxidant capacity of Chinese Holstein cows than SFC.

### 4.3 Effect of feeding HMC on intestinal microflora of Chinese Holstein cows

Gut microbiota diversity is associated with intestinal health and disease resistance (32). From this study, the GSFY group showed slightly lower Chao1 and Simpson indices. Paula Almeida et al. (33) reported that HMC fermentation significantly increased acetate content, the resulting short-chain fatty acids (SCFAs) such as acetate and propionate can lower intestinal pH, suppress sensitive microbial groups, and promote the proliferation of fibrolytic bacteria, leading to a mild decrease in diversity and an increase in the abundance of dominant taxa. In addition, Mary et al. (34) indicated that dietary fiber intake is significantly correlated with Alpha diversity, SCFAs produced from fiber fermentation help reduce pH and maintain the stability of commensal bacteria, thereby supporting microbial diversity. Beta diversity analysis showed significant separation in PCoA of gut microbiota between GSFY and YPFY groups. Wang et al. (35) confirmed different starch sources reshape gut microbiota by altering VFAs production patterns, consistent with this study. Thus, feeding HMC significantly changes gut microbiota structure but not overall diversity in Chinese Holstein cows.

In the GSFY group, *Bacteroidota* abundance significantly increased, while *Firmicutes_A* and *Actinobacteriota* significantly decreased. *Bacteroidota* plays a key role in dairy cow nutrient metabolism by degrading plant polysaccharides (36). HMC fermentation produces SCFAs that reduce intestinal pH; *Bacteroidota* tolerates acidic environments well, while *Firmicutes_A* and *Actinobacteriota* are pH-sensitive so their growth is inhibited, leading to increased *Bacteroidota* abundance but decreased *Firmicutes_A* and *Actinobacteriota* abundance (37). In the GSFY group, the abundances of *Paraprevotella* and *Succinivibrio* were significantly enriched, whereas those of *Prevotella* and *Faecousia* were markedly reduced. Shang et al. (23) noted that HMC increases starch availability and utilization by microbes; *Paraprevotella* likely grows by utilizing the elevated SCFAs produced during HMC fermentation. Xue et al. (38) reported that *Succinivibrio* abundance correlates positively with propionate production. HMC fermentation generates substantial propionate and succinate, providing sample substrates for *Succinivibrio* growth. Shinkai et al. (39) indicated that rapid fermentation of high-starch substrates promotes propionate accumulation, *Prevotella* preferentially ferments lactate rather than succinate, placing it at a competitive disadvantage and leading to its decreased abundance.

Compared with the YPFY group, the metabolic activities of *Ruminobacter* and *Succinivibrio* were significantly enhanced in the GSFY group, while those of *Prevotella* and *Faecousia* were markedly reduced. Li et al. (37) showed that high-starch diets enrich starch-degrading bacteria such as *Ruminobacter* and activate the lactate-derived acrylate pathway to promote propionate production. This aligns with the elevated propionate levels and enhanced *Ruminobacter* activity observed with HMC feeding. In addition, Xue et al. (38) found that *Succinivibrio* plays a key role in microbial interactions, utilizing hydrogen to produce succinate, which may improve propionate synthesis and feed efficiency. In contrast, Kong et al. (40) indicated that *Prevotella*, although important in propionate metabolism, exhibited downregulated relevant pathways, reduced substrate availability, and intensified microbial competition in this trial, thereby suppressing its metabolic activity. In summary, feeding HMC can modulate the abundance and metabolic activity of dominant bacterial groups, influencing metabolic function in dairy cows.

### 4.4 Correlation analysis of feeding HMC on intestinal microorganisms and milk composition of Chinese Holstein cows

In this study, milk yield was negatively correlated with *Succinivibrio* and *WG-1*, but positively correlated with *Treponema_D*. *Succinivibrio* participates in succinate metabolism and can generate propionate precursors (41), however, its excessive proliferation increases the propionate-to-acetate ratio, competes for carbohydrate substrates, reduces fiber degradation and acetate production, and ultimately affects milk yield. Lactose was negatively correlated with *Cryptobacteroides* and *Succiniclasticum*, while positively correlated with *Faecousia*, *Treponema_D*, and *Succinivibrio*. *Treponema* can supply glucose precursors for lactose synthesis, but its metabolites may be prioritized for energy supply, thus reducing lactose content (42). Meanwhile, *Succinivibrio* influences energy metabolism through the succinate-propionate pathway, which may be negatively associated with lactose synthesis (43). Milk fat was negatively correlated with *Prevotella*, *Treponema_D*, and *Succinivibrio*, but positively correlated with *Cryptobacteroides* and *Succiniclasticum*. In the present study, a HMC diet characterized by low NDF and high starch content significantly reduced the abundance of *Prevotella* and led to a decrease in the propionate/acetate ratio, which is consistent with the core finding reported by Zhang et al. (44) that *Prevotella* is involved in ruminal VFAs metabolism. Acetate is a major precursor for milk fat synthesis (45), and HMC contains higher levels of propionate, both contributing to the elevated milk fat percentage in the GSFY group. Yang et al. (46) indicated that *Succiniclasticum* converts succinate to propionate, and changes in its activity may affect milk fat percentage. Milk protein was negatively correlated with *Faecousia*, *Cryptobacteroides* and *Succinivibrio*. Amin et al. (47) found that the abundance of *Succinivibrio* was high in the low milk protein group, which was consistent with this study. Non - milk fat solids were negatively correlated with *Treponema_D* and *Succinivibrio*. Granja et al. (48) confirmed that *Succinivibrio* affects production performance by regulating VFAs and energy metabolism. The enrichment of *Succinivibrio* in GSFY group may change the intestinal environment and affect the production of non-milk fat solids.

## 5 Conclusion

Feeding HMC maintains milk yield, increases milk fat percentage, enhances serum antioxidant enzyme activities, alters gut microbiota structure with increased *Bacteroidota* abundance, and regulates milk component synthesis via gut microbiota modulation. In conclusion, this study provides theoretical basis for HMC application in dairy diets and supports research on improving milk quality and antioxidant capacity.

## Data Availability

The original contributions presented in the study are publicly available. This data can be found here: Figshare, https://doi.org/10.6084/m9.figshare.31885540.

## Supplementary material

SUPPLEMENTARY TABLE S1 Alpha diversity indices of gut microbiota in Chinese Holstein cows fed SFC (YPFY) or HMC (GSFY).

## References

[1] Meng JT Huang WZ Chen SW Zhang RN Ma JY. Effect of wet storage of whole cob corn on performance and feed cost of dairy cows. Gansu Anim Husb Vet Med. (2020) 50:48–52. doi: 10.15979/j.cnki.cn62-1064/s.2020.09.016

[2] Clark JH Harshbarger KE. High-moisture corn versus dry corn in combination with either corn silage or Hay for lactating cows. J Dairy Sci. (1972) 55:1474–80. doi: 10.3168/jds.S0022-0302(72)85697-2

[3] National Research Council. Nutrient requirements of dairy cattle. 7th ed. Washington, DC: National Academies Press (2001).

[4] Dai ZX Li Z Liao CX Hu TT Liang Y Zhao Q . Analysis of key points in making HMC silage. China Feed. (2022) 17:111–6. doi: 10.15906/j.cnki.cn11-2975/s.20221720

[5] Zhou Y Drouin Y Lafreniere C. Effect of temperature(5-25°C) on epiphytic lactic acid bacteria populations and fermentation of whole-plant corn silage. J Appl Microbiol. (2016) 121:657–71. doi: 10.1111/jam.13198 27271320

[6] Knowlton KF Glennbp RA Erdman P. Performance, ruminal fermentation, and site of starch digestion in early lactation cows fed corn grain harvested and processed differently. J Dairy Sci. (1998) 81:1972–84. doi: 10.3168/jds.S0022-0302(98)75771-6 9710767

[7] Firkins JL Eastridge ML St-Pierre NR Noftsger SM. Effects of grain variability and processing on starch utilization by lactating dairy cows. J Anim Sci. (2001) 79:E218–38.

[8] Han JY Song LH Zhang CX Zhang JM Liu KY. Effects of feeding high-moisture wet-storage corn on dairy cow performance and feeding cost. Feed Industry. (2018) 39:19–22. doi: 10.2527/jas2001.79E-SupplE218x

[9] Liu CL Li ZQ Zhang F Jiang WB Sun S Xu Y . Effects of soybean flavonoids and genistein on serum biochemical and blood rheological indices in dairy cows. Chinese J. Vet. Med. (2009) 4:460–5. doi: 10.13302/j.cnki.fi.2018.17.004

[10] Williams KJ Ward MP Dhungyel O van Breda L. Relative sensitivity of *Escherichia coli* O157 detection from bovine feces and rectoanal mucosal swabs. J Food Prot. (2014) 77:972–6. doi: 10.4315/0362-028X.JFP-13-500, 24853520

[11] Klindworth A Pruesse E Schweer T Peplies J Quast C Horn M . Evaluation of general 16S ribosomal RNA gene PCR primers for classical and next-generation sequencing-based diversity studies. Nucleic Acids Res. (2013) 41:e1. doi: 10.1093/nar/gks808., 22933715 PMC3592464

[12] Bolyen E Rideout JR Dillon MR Bokulich NA Abnet CC al-Ghalith GA . Reproducible, interactive, scalable and extensible microbiome data science using QIIME 2. Nat Biotechnol. (2019) 37:852–7. doi: 10.1038/s41587-019-0209-9, 31341288 PMC7015180

[13] Quast C Pruesse E Yilmaz P Gerken J Schweer T Yarza P . The SILVA ribosomal RNA gene database project: improved data processing and web-based tools. Nucleic Acids Res. (2013) 41:D590–6. doi: 10.1093/nar/gks1219., 23193283 PMC3531112

[14] Eun JS Kelley AW Neal K Young AJ Hall JO. Effects of altering alfalfa hay quality when feeding steam-flaked versus HMC grain on ruminal fermentation and lactational pertational of dairy cows. J Dairy Sci. (2014) 97:7833–43. doi: 10.3168/jds.2014-8425 25262185

[15] Russell JB O'Conno JD Fox DG O'Connor JD Van Soest PJ Sniffen CJ. A net carbohydrate and protein system for evaluating cattle diets: I. Ruminal fermentation. J Anim Sci. (1992) 70:3551–61. doi: 10.2527/1992.70113551x, 1459918

[16] Yi XB. Factors affecting milk fat rate of dairy cows and ways to improve it. Modern Anim Husb Sci Technol. (2020) 11:46–8. doi: 10.19369/j.cnki.2095-9737.2020.11.024

[17] Chandler PT Miller CN Jahn E. Feeding value and nutrient preservation of high moisture corn ensiled in conventional silos for lactating dairy cows. J Dairy Sci. (1975) 58:682–8. doi: 10.3168/jds.S0022-0302(75)84628-5, 1141478

[18] Shang SL Li T Li JJ Hu TT Chen YD Li D . Effects of HMC with shaft on performance, serum biochemical indexes, immune function and antioxidant capacity of dairy cows. J Anim Nutr. (2023) 35:5226–35. doi: 10.12418/CJAN2023.483

[19] Mizrahi I Wallace RJ Moraïs S. The rumen microbiome: balancing food security and environmental impacts. Nat Rev Microbiol. (2021) 19:553–66. doi: 10.1038/s41579-021-00543-6, 33981031

[20] Russell JB Cotta MA Dombrowski DB. Ruminal bacterial competition in continuous culture: *Streptococcus bovis* versus *Megasphaera elsdenii*. Appl Environ Microbiol. (1981) 41:1394–9. doi: 10.1128/aem.41.6.1394-1399.1981 16345793 PMC243929

[21] Fan Y Li SL Kong FL Wang W. Factors influencing the nutritional value of high-moisture corn and its application in dairy production. Chin J Anim Nutr. (2022) 34:68673–6880. doi: 10.3969/j.issn.1006-267x.2022.11.007

[22] Voelker HH Schingoethe DJ Drackley JK Clark AK. High-moisture corn preserved by different methods for lactating cows. J Dairy Sci. (1985) 68:2602–7. doi: 10.3168/jds.S0022-0302(85)81143-7

[23] Shang S Li J Zhang W Zhang X Bai J Yang Z . Impact of high-moisture ear corn on antioxidant capacity, immunity, rumen fermentation, and microbial diversity in pluriparous dairy cows. Fermentation. (2024) 10:44. doi: 10.3390/fermentation10010044

[24] Amar Abdoul-Aziz SK Abdoul-Aziz SKA Zhang Y Wang J. Milk odd and branched chain fatty acids in dairy cows: a review on dietary factors and its consequences on human health. Animals. (2021) 11:3210. doi: 10.3390/ani11113210, 34827941 PMC8614267

[25] Rojas-Garduño MA Rojas-Garduño M d l Á Balocchi O Vicente F Pulido R. Effect of supplementation with cracked wheat or high moisture corn on milk fatty acid composition of grazing dairy cows. Chil J Agric Res. (2018) 78:96. doi: 10.4067/S0718-58392018000100096

[26] Alexander VP Sergey ID. Cardiolipin, perhydroxyl radicals, and lipid peroxidation in mitochondrial dysfunctions and aging. Oxidative Med Cell Longev. (2020) 2020:1323028. doi: 10.1155/2020/1323028, 32963690 PMC7499269

[27] Ighodaro OM Akinloye OA. First line defence antioxidants-superoxode (SOD), catalase (CAT) and glutathione peroxidase (GPX): their fundamental role in the entire antioxidant defence grid. Alexandria J Med. (2018) 54:287–93. doi: 10.1016/j.ajme.2017.09.001

[28] Cheryl LF Lisa MS Oury TD. Extracellular superoxide dismutase in biology and medicine. Free Radic Biol Med. (2003) 35:236–56. doi: 10.1016/s0891-5849(03)00275-2, 12885586

[29] Yang LL Huang MS Huang CC Wang TH Lin MC Wu CC . The association between adult asthma and superoxide dismutase and catalase gene activity. Int Arch Allergy Immunol. (2011) 156:373–80. doi: 10.1159/000324448, 21829032

[30] Albornoz RI Sordillo LM Contreras GA Nelli R Mamedova LK Bradford BJ . Diet starch concentration and starch fermentability affect markers of inflammatory response and oxidant status in dairy cows during the early postpartum period. J Dairy Sci. (2020) 103:352–67. doi: 10.3168/jds.2019-16398, 31733858

[31] Kullisaar KT Songisepp E Aunapuu M Kullisaar T Kilk K Arend A . Complete glutathione system in probiotic *Lactobacillus fermentum* ME-3. Appl Biochem Microbiol. (2010) 46:481–6. doi: 10.1134/s0003683810050030, 21058502

[32] Renata PSM Joanis TZ Luciano N da Silva-Marques RP Zervoudakis JT Nakazato L . Quantitative qPCR analysis of ruminal microorganisms in beef cattle grazing in pastures in the rainy season and supple mented with different protein levels. Curr Microbiol. (2018) 75:1025–32. doi: 10.1007/s00284-018-1484-2 29594405

[33] Paula Almeida CE Carvalho-Estrada P d A Fernandes J da Silva ÉB Tizioto P Paziani S d F . Effects of hybrid, kernel maturity, and storage period on the bacterial community in high-moisture and rehydrated corn grain silages. Syst Appl Microbiol. (2020) 43:126131. doi: 10.1016/j.syapm.2020.126131 32866836

[34] Kable ME Chin EL Storms D Lemay DG Stephensen CB. Tree-based analysis of dietary diversity captures associations between Fiber intake and gut microbiota composition in a healthy US adult cohort. J Nutr. (2022) 152:779–88. doi: 10.1093/jn/nxab430, 34958387

[35] Wang X Wang DD Nian F Tang DF Wang WM Ma ZY . Effects of diets with different starch sources on intestinal digestive enzyme activities, cecal volatile fatty acid concentrations, and microbial flora in broiler chickens. Chin J Anim Nutr. (2021) 33:285–96. doi: 10.3969/j.issn.1006-267x.2021.01.029

[36] Jenna RW Todd RC Jeferson ML Williamson JR Callaway TR Lourenco JM . Characterization of rumen, fecal, and milk microbiota in lactating dairy cows. Front Microbiol. (2022) 13:984119. doi: 10.3389/fmicb.2022.984119, 36225385 PMC9549371

[37] Li QS Wang R Ma ZY Zhang XM Jiao JZ Zhang ZG . Dietary selection of metabolically distinct microorganisms drives hydrogen metabolism in ruminants. ISME J. (2022) 16:2535–46. doi: 10.1038/s41396-022-01294-9, 35931768 PMC9562222

[38] Xue MY Xie YY Zhong Y Ma XJ Sun HZ Liu JX. Integrated meta-omics reveals new ruminal microbial features associated with feed efficiency in dairy cattle. Microbiome. (2022) 10:32. doi: 10.1186/s40168-022-01228-9, 35172905 PMC8849036

[39] Shinkai T Ikeyama N Kumagai M Ohmori H Sakamoto M Ohkuma M . Prevotella lacticifex sp. nov., isolated from the rumen of cows. Int J Syst Evol Microbiol. (2022) 72:278. doi: 10.1099/ijsem.0.005278, 35254232

[40] Kong F Wang S Zhang Y Li C Dai D Guo C . Rumen microbiome associates with postpartum ketosis development in dairy cows: a prospective nested case–control study. Microbiome. (2025) 13:69. doi: 10.1186/s40168-025-02072-3, 40057813 PMC11889851

[41] Xue MY Sun HZ Wu XH Guan LL Liu JX. Assessment of rumen bacteria in dairy cows with varied milk protein yield. J Dairy Sci. (2019) 102:5031–41. doi: 10.3168/jds.2018-15974, 30981485

[42] Wang S Kong FL Dai DW Li C Hao Y Wang E . Deterministic succession patterns in the rumen and fecal microbiome associate with host metabolic shifts in peripartum dairy cattle. GigaScience. (2025) 14:042. doi: 10.1093/gigascience/giaf042, 40388308 PMC12087452

[43] Wang D Tang LG Chen L Tang G Yu J Chen J . Multi-omics revealed the long-term effect of ruminal keystone bacteria and the microbial metabolome on lactation performance in adult dairy goats. Microbiome. (2023) 11:215. doi: 10.1186/s40168-023-01652-5, 37773207 PMC10540338

[44] Zhang XJ Wang JZ. Effects of dietary neutral detergent fiber level on the structure and composition of rumen bacteria in goats. Chin J Anim Nutr. (2018) 30:1377–86. doi: 10.3969/j.issn.1006-267x.2018.04.020

[45] Guo YM Liu Y Wu R Yan SM Zhao YL Guo XY. Effects of the interaction between vitamin a and acetic acid on the expression of genes related to milk component synthesis in bovine mammary epithelial cells. Sci Agric Sin. (2023) 56:4344–58. doi: 10.3864/j.issn.0578-1752.2023.21.016

[46] Yang JH Li YF Sun MK Yang J Li Y Sun M . Understanding the differences in rumen bacteria and their impact on dairy cows' production performance: a review. Anim Nutr. (2025) 22:6. doi: 10.1016/j.aninu.2025.04.006, 40896480 PMC12391802

[47] Amin AB Zhang L Zhang J Mao S. Metagenomics analysis reveals differences in rumen microbiota in cows with low and high milk protein percentage. Appl Microbiol Biotechnol. (2023) 107:4887–902. doi: 10.1007/s00253-023-12620-2, 37306708

[48] Granja-Salcedo YT Duarte MJ Carneiro SV Duarte Messana J de Carneiro Souza V Lino Dias AV . Effects of partial replacement of maize in the diet with crude glycerin and/or soyabean oil on ruminal fermentation and microbial population in Nellore steers. Br J Nutr. (2017) 118:651–60. doi: 10.1017/S0007114517002689, 29185932

